# The *Medical Counselor* and the I. H. A.

**Published:** 1883-02

**Authors:** 


### THE "MEDICAL COUNSELOR" AND THE I. H. A.

In the December 1st issue of the *Counselor* the editor wastes a good deal of time and space in a criticism of the International Hahnemannian Association. After some preliminary remarks, which might be regarded as a kind of preamble to a resolution, he says: "The conception of the I. H. A. dates back to the day when its leaders found themselves in too hopeless a minority in the American Institute to have their own way, and when no more honors were to be gained."

This is assertion without proof. In fact, there is not a word of truth in it. When did the leaders of the I. H. A. aspire to honors in the A. I.? Never. In fact, some of them are very doubtful if it has any honors to confer. The critic also says: "So far as the A. I. is concerned, its record shows many a sad, though not one *fatal* mistake." It would seem the writer is not well posted as to its record. What kind of mistake would he call the striking out of the qualification for membership the word "Homœopathy," during its session at Niagara Falls in 1874? What kind of mistake, the passing of the Dudley resolution at Indianapolis? Or how many such blunders does he think it would require to constitute "one *fatal* mistake"? He says "the claims of the minority in the A. I. have ever been treated with consideration." What kind of consideration? Were Hahnemann himself a member of that body to-day, and to write and advocate the sentiments of the *Organon*, if he would not be insulted by some of its members it would be his name rather than his words that would protect him. Very rarely for the past ten years, and *never* for the past five, has such a paper as he would have fully indorsed been read at one of its meetings without the writer being ridiculed and his paper being unjustly criticised, and for no other reason than that it was the God's truth as Hahnemann taught it. How was it at the last meeting, when a paper was read, written by as pure a homœopathist as ever lived, and one, too, the latchet of whose shoes many of the leaders of the Institute are not worthy to unloose—Henry N. Guernsey? Not one word was said in its favor, but speech after speech made by way of ridicule, one speaker going so far as to say he would not say the writer lied when he stated that certain diseases were more successfully treated with potencies at and above the 30th, but he intimated that he did not tell the truth. And this is now the treatment that such papers invariably receive at the hands of the Institute. One active member says in the journals what he would be sustained in saying in any of its meetings, that "Every day we listen to reports of these nondescript dynamic cases at the meetings of our societies and publish them as homœopathic, without protest, we are acting a *lie*." And yet we are asked to endure all this kindly; denounced if we retaliate, and accused of using "bitter and undignified language." If a writer who is so free to give the *lie* and other "bitter and undignified language," or even the editor of the *Counselor*, can see his own portrait in the story told by the President of the I. H. A. of the man who was frightened at his own shadow, he has only to be more careful in the selection of his language. If H. M. Paine or Pemberton Dudley has not "taken advantage of the most exalted official position in the A. I. to speak of the members of the I. H. A. in the bitter and undignified manner in which the President of the latter body in his address referred to H. M. Paine and those who share his views," it is probably only because he does not occupy any such position. But the late occupant of that "exalted official position" in the A. I. "in his address" did speak of the members of the I. H. A. as "bottle washers," and the Institute voted to have five thousand copies of the address printed, plagiarism and all, as an evidence of its indorsement of the sentiments it contained, and had there been any precedent for his re-election he would probably have been elected again to that "exalted official position," and when the Dudley resolution was passed, with only one dissenting vote, this same President, occupying this "exalted official position," stated that he was sorry the vote was not unanimous. And yet the pure, sensitive soul of the editor of the *Counselor* has no words of censure for all this—Oh! no, not he! He seems to think it his holy mission to point out the "drift of the International Hahnemannian Association," but he can see no drift of the A. I. There is such a thing as holding an object too near the eyes to see it, but he thinks he has about the right focus on the I. H. A. We would suggest that he give his glass (of course, we mean eye-glass) another twist, and see if he can discern any "drift" to the A. I. He appears to think, however, that it still needs the assistance of these do-nothing Internationals, for he says: "Our school has reached a stage where it needs cool and deliberate leadership and concert of action." Very likely. Are there breakers ahead? Who is running the craft, and who has put it in this critical position? Certainly not any of the members of the I. H. A.

This critic still further affirms that this Association "has done nothing save the cultivation of outspoken bigotry and arrogance on the part of its leaders." This "bigotry and arrogance," we suppose, consists in upholding and defending the *Organon* against the line of policy mapped out by the leaders of the A. I. And here, again, the editor's focus is at fault, as he can see no "bigotry and arrogance" in his own criticism. He wonders if it be probable that the I. H. A. will ever "bring about any remarkable reforms in homœopathic medicine." He is asking too much if he expects this. Reforms on natural laws are not easily accomplished. It takes such journals as the *Counselor* and such reformers as some of the leaders of the A. I. to bring this about. They are the re-formers (backward); the *Organon* is good enough for us. He says that "not one trace of any drug-proving or any original work in any one direction can be found in the records of said Association." This would serve his purpose better if it were true, but, unfortunately, it is not. If he will look at the proceedings of the first meeting of the I. H. A., as published in the July, 1881, number of THE HOMŒOPATHIC PHYSICIAN, he will find as good a proving of Amm. carb. by a lady member of the Association as can be found anywhere. This lady also, at great risk, proved Medorrhin. This proving, which is quite voluminous, will soon be published; but being of one of the nosodes—and therefore, according to the *Counselor*, a "non-Hahnemannian nonsense," notwithstanding Hahnemann and his followers ever since have used nosodes—it will not count in the *Counselor's* materia medica.

Now, as the members of the A. I. outnumber those of the I. H. A. ten to one, and have been well organized for over forty years, they should, at least, in the past two or three years have proven twenty-five or thirty medicines. But have they done this? If so, "not one trace can be found in the records of said Association." They have given us fragmentary provings of a few drugs, two of the most valuable of which, according to the report of the chairman of the Bureau of Materia Medica and Provings for 1881, were furnished by two members of the I. H. A., H. C. Allen and Wm. H. Leonard, so that we cannot see wherein the *Counselor* has any great cause for boasting. It predicts the dissolution of the International Association, because "its small membership represents as many shades of belief as can be found in the A. I. Does he think this diversity of belief portends divisions in the A. I.? Two of its members who seem to be greatly exercised about the best way to dispose of " dynamism" in that body differ materially in regard to it. One says it is non-homœopathic, but that Hahnemann taught it. He fails to tell us, however, who taught Homœopathy before Hahnemann. The other says it is non-homœopathic and that Hahnemann did not teach it (N. Y. *Medical Times*, Jan., 1883). Has the I. H. A. anything more difficult on its hands than it would be to reconcile these absurdities with truth and history? But our good editor says: "So far as the *Medical Counselor* is concerned, it will ever be ready to expose blunders wherever found." Well, now, we are glad, and propose that he go to work on the above "blunders" at once. He denounces the President of the I. H. A. for intimating a withdrawal from the A. I. No member of the I. H. A. would have any thoughts of withdrawing his connection with it did it manifest a disposition to carry out the original intent of its founders, so long, in short, as it does not withdraw from them and from Homœopathy, and even steal the name and the livery of Heaven to serve the devil in. He also predicts that those members of the I. H. A. who went into it "with clean hands and hearts" will be forced to withdraw from it.

> O flesh! flesh! how art thou fishified,
> Springs to catch woodcocks.

Does he know of any gudgeon ready to swallow his bait? We would have him understand that the Internationals are men who are not usually identified with a failure, that they have resolved themselves into a vigilance committee and are on the track of traitors—of those physicians and all who fraternize with them who, to use the language of the *New York Hospital Gazette*, "have taken the lead in firing upon and hauling down their own flag." If our protests and remonstrances will not be heeded by that body, but it allows itself to be controlled and contaminated by them; if, in short, all the crude elements in the medical world are to be invited to come in under the name of Homœopathy; if those who see proper to prescribe remedies above the 12th centesimal potency are to be styled *dynamists*, while they who use the 11th are to be classed as homœopaths, which seems now to be the latest device of these hard-working members, who, according to the *Counselor*, have done, and are still doing so much for the cause of Homœopathy;—if all this is to be brought about, and the following preamble and resolution, which one of these members proposes, shall be adopted by the A. I. of Homœopathy, then we will make a fight for the name, and our battle-cry will be—

"The Organon, Homœopathy, and Victory!" which, like the heart of Bruce, will ever carry dismay into the ranks of the traitors. But here is the resolution. How does the *Counselor* like it?

"WHEREAS, Long experience, conducted by thousands of homœopathists in the use of high potencies, has conclusively proved their unscientific, untrustworthy, and non-homœopathic character; therefore

"*Resolved*, That there be established a department of dynamic medicine, the members of which shall be annually appointed in the same manner as those of other bureaux, to which shall be referred all reports of cases presented to the Society or gathered from other sources alleged to have been cured by attenuations higher than the 12th potency."

Will the good *Counselor*, "ever ready to expose blunders wherever found," take any exception to this? Or will it think this is doing something "for the good of the profession at large"? In conclusion, we would just say, in the language of the *Counselor*, "It will require much wire-pulling, much shrewdness, and considerable executive ability to keep up even a semblance of harmony in so mixed a family," and we would advise the editor to look after these matters in the A. I. and let the affairs of the I. H. A. alone. Should his assistance be needed, he will be so informed, but at present he need not apply, as there is no vacancy.

AN INTERNATIONAL.

